# Supplementary material for: Disease burden of asbestos-related diseases in China (1990–2023) based on GBD estimates: A call for stronger labor protection laws
Source: PLoS One. 2026 May 18;21(5):e0349392. doi: 10.1371/journal.pone.0349392 (PMC13183203; doi:10.1371/journal.pone.0349392)
Supplement: S1 Table — (DOCX) [file pone.0349392.s001.docx]

S1 Table. Joinpoint regression of asbestosis in China, 1990-2023

| Indicators | Period | APC (95% CI) | AAPC (95% CI) |
| --- | --- | --- | --- |
| ASPRs | 1990-1994 | 9.6394 (7.4227, 11.9018) * | 0.5751 (0.2084, 0.9431) * |
|  | 1994-2000 | 2.1769 (0.6467, 3.7302) * |  |
|  | 2000-2023 | -1.3306 (-1.4734, -1.1876) * |  |
| ASIRs | 1990-1994 | 4.7716 (2.6848, 6.9008) * | 0.3300 (-0.0198, 0.6812) |
|  | 1994-2000 | 1.8778 (0.4570, 3.3188) * |  |
|  | 2000-2023 | -0.8198 (-0.9588, -0.6807) * |  |
| ASMRs | 1990-1994 | 0.2681 (-2.2277, 2.8275) | 1.1891 (0.7280, 1.6523) * |
|  | 1994-2004 | 8.1872 (7.5312, 8.8472) * |  |
|  | 2004-2015 | -4.669 (-4.9874, -4.3495) * |  |
|  | 2015-2019 | -0.5975 (-2.6033, 1.4496) |  |
|  | 2019-2023 | 3.6239 (2.0257, 5.2472) * |  |
| ASDRs | 1990-2004 | 4.7941 (4.4966, 5.0924) * | 0.8802 (0.5922, 1.1690) * |
|  | 2004-2015 | -3.5718 (-3.8613, -3.2813) * |  |
|  | 2015-2019 | -0.9968 (-2.6506, 0.6852) |  |
|  | 2019-2023 | 1.8614 (0.5476, 3.1924) * |  |

* Indicates that the APC is statistically significant (P < 0.05).

APC: Annual Percent Change; AAPC: Average Annual Percent Change; CI: Confidence Interval; ASPRs: Age-Standardized Prevalence Rates; ASIRs: Age-Standardized Incidence Rates; ASMRs: Age-Standardized Mortality Rates; DALY: Disability-Adjusted Life Year; ASDRs: Age-Standardized DALY Rates.
