# Supplementary material for: Disease burden of asbestos-related diseases in China (1990–2023) based on GBD estimates: A call for stronger labor protection laws
Source: PLoS One. 2026 May 18;21(5):e0349392. doi: 10.1371/journal.pone.0349392 (PMC13183203; doi:10.1371/journal.pone.0349392)
Supplement: S2 Table — (DOCX) [file pone.0349392.s002.docx]

S2 Table. Joinpoint regression of asbestos-related cancers in China, 1990-2023

| Indicators | Period | APC (95% CI) | AAPC (95% CI) |
| --- | --- | --- | --- |
| *Mesothelioma* |  |  |  |
| ASMRs | 1990-2004 | -0.3209 (-0.6340, -0.0069) * | 0.1674 (-0.2013, 0.5374) |
|  | 2004-2010 | 3.7756 (2.4426, 5.1261) * |  |
|  | 2010-2020 | -2.1049 (-2.5495, -1.6584) * |  |
|  | 2020-2023 | 3.0644 (0.3388, 5.8641) * |  |
| ASDRs | 1990-2004 | -0.4461 (-0.7412, -0.1501) * | 0.1469 (-0.2081, 0.5031) |
|  | 2004-2010 | 4.3246 (3.0623, 5.6022) * |  |
|  | 2010-2020 | -2.3343 (-2.7581, -1.9087) * |  |
|  | 2020-2023 | 3.1560 (0.4537, 5.9311) * |  |
| *Tracheal, bronchus, and lung cancer* |  |  |  |
| ASMRs | 1990-2004 | 1.0556 (0.8103, 1.3015) * | 0.7519 (0.1899, 1.3172) |
|  | 2004-2009 | 5.0107 (3.4039, 6.6424) * |  |
|  | 2009-2017 | -1.7862 (-2.4114, -1.1570) * |  |
|  | 2017-2020 | -4.5828 (-9.2222, 0.2936) |  |
|  | 2020-2023 | 4.8006 (2.0145, 7.6629) * |  |
| ASDRs | 1990-2004 | 0.5263 (0.2884, 0.7648) * | 0.4148 (-0.1388, 0.9714) |
|  | 2004-2009 | 4.6781 (3.0989, 6.2815) * |  |
|  | 2009-2017 | -2.0711 (-2.6921, -1.4461) * |  |
|  | 2017-2020 | -4.6108 (-9.2020, 0.2126) |  |
|  | 2020-2023 | 4.9014 (2.1562, 7.7203) * |  |
| *Laryngeal cancer* |  |  |  |
| ASMRs | 1990-2004 | -1.9040 (-2.1696, -1.6377) * | -0.8634 (-1.2477, -0.4777) * |
|  | 2004-2010 | 3.5276 (2.2548, 4.8163) * |  |
|  | 2010-2020 | -3.2336 (-3.7383, -2.7263) * |  |
|  | 2020-2023 | 3.5154 (0.3620, 6.7680) * |  |
| ASDRs | 1990-2005 | -2.1604 (-2.3889, -1.9313) * | -0.9922 (-1.7073, -0.2719) * |
|  | 2005-2008 | 5.7163 (-0.0500, 11.8154) |  |
|  | 2008-2011 | 0.3417 (-4.6867, 5.6355) |  |
|  | 2011-2020 | -3.1836 (-3.7630, -2.6007) * |  |
|  | 2020-2023 | 3.8291 (0.6738, 7.0833) * |  |
| *Ovarian cancer* |  |  |  |
| ASMRs | 1990-2001 | -0.7689 (-0.9696, -0.5677) * | -0.3265 (-0.8042, 0.1535) |
|  | 2001-2005 | -3.8111 (-5.2193, -2.3820) * |  |
|  | 2005-2011 | 3.2158 (2.5520, 3.8839) * |  |
|  | 2011-2014 | -4.4326 (-7.1044, -1.6839) * |  |
|  | 2014-2017 | 0.7768 (-2.1228, 3.7623) |  |
|  | 2017-2020 | -2.5453 (-5.3515, 0.3441) |  |
|  | 2020-2023 | 4.5207 (2.9628, 6.1022) * |  |
| ASDRs | 1990-2001 | -1.1605 (-1.3831, -0.9374) * | -0.5247 (-1.0443, -0.0024) * |
|  | 2001-2005 | -3.5328 (-5.0876, -1.9525) * |  |
|  | 2005-2011 | 2.7640 (2.0391, 3.4941) * |  |
|  | 2011-2014 | -4.4349 (-7.3267, -1.4528) * |  |
|  | 2014-2017 | 0.4088 (-2.7165, 3.6344) |  |
|  | 2017-2020 | -2.5486 (-5.6157, 0.6182) |  |
|  | 2020-2023 | 4.6508 (2.9489, 6.3809) * |  |

* Indicates that the APC is statistically significant (P < 0.05).

APC: Annual Percent Change; AAPC: Average Annual Percent Change; CI: Confidence Interval; ASMRs: Age-Standardized Mortality Rates; DALY: Disability-Adjusted Life Year; ASDRs: Age-Standardized DALY Rates.
