# Supplementary material for: Disease burden of asbestos-related diseases in China (1990–2023) based on GBD estimates: A call for stronger labor protection laws
Source: PLoS One. 2026 May 18;21(5):e0349392. doi: 10.1371/journal.pone.0349392 (PMC13183203; doi:10.1371/journal.pone.0349392)
Supplement: S3 Table — (DOCX) [file pone.0349392.s003.docx]

**S3 Table. Legal Regulations on Protective Requirements for Asbestos Operations in China and Major Industrialized Countries in Europe and North America**

| Country | Prohibition of Asbestos Production and Use | 8-Hour TWA (f/cm³) | Mandatory Use of HEPA | Minimum Respirator/Mask Level Requirement | Mandatory Frequency of Environmental Asbestos Concentration Testing |
| --- | --- | --- | --- | --- | --- |
| China^1^ | No | 0.8 | No | None | Principle-based only; no mandated testing frequency |
| United States^2^ | No | 0.1 | Yes | N100/P100 | Every 3–6 months depending on exposure results |
| United Kingdom^3^ | Yes | 0.1 | Yes | FFP3 | Periodic monitoring; continuous monitoring for high-risk work |
| Germany^4^ | Yes | 0.1 | Yes | FFP3 / Full-face Respirator | Regular monitoring; continuous assessment in high-risk demolition |
| France^5^ | Yes | 0.01 | Yes | FFP3 / P3 Filter Cartridge | At least every 6 months; frequent/continuous monitoring for high-risk work |

1. National Health Commission of the People’s Republic of China. (2019). Occupational exposure limits for hazardous agents in the workplace – Part 1: Chemical hazardous agents (GBZ 2.1-2019).

2. Occupational Safety and Health Administration. (n.d.). Asbestos (29 CFR 1910.1001). U.S. Department of Labor. https://www.osha.gov/laws-regs/regulations/standardnumber/1910/1910.1001

3. UK Government. (2012). Control of Asbestos Regulations 2012 (SI 2012/632). https://www.legislation.gov.uk/uksi/2012/632/contents/made

4. Federal Ministry of Labour and Social Affairs. (2019). Technical Rules for Hazardous Substances: TRGS 519 – Asbestos: Demolition, reconstruction, and maintenance work. https://www.baua.de

5. French Ministry of Labour. (n.d.). Labour Code – Provisions on asbestos exposure and worker protection. https://www.legifrance.gouv.fr

TWA: Time-Weighted Average; HEPA: High-Efficiency Particulate Air; FFP3: Filtering Face Piece Class 3.
